# The rheumatoid arthritis shared epitope increases cellular susceptibility to oxidative stress by antagonizing an adenosine-mediated anti-oxidative pathway

**DOI:** 10.1186/ar2111

**Published:** 2007-01-25

**Authors:** Song Ling, Zhanguo Li, Olga Borschukova, Liqun Xiao, Paul Pumpens, Joseph Holoshitz

**Affiliations:** 1 Department of Internal Medicine, University of Michigan, 1150 W. Medical Center Dr., 5520 MSRB I, Ann Arbor, MI 48109-0680, USA; 2 Department of Rheumatology, Beijing Medical University, Beijing, 11 S. Xizhimen Blvd, Beijing, 100044, The People's Republic of China; 3 Biomedical Research and Study Center, University of Latvia, Ratsupites 1, Riga, LV-1067, Latvia

## Abstract

We have recently demonstrated that the rheumatoid arthritis (RA) shared epitope (SE) acts as a ligand that triggers nitric oxide (NO) signaling in opposite cells. Given the known pro-oxidative effect of NO and the proposed role of oxidative stress in the pathogenesis of RA, this study explores whether SE-triggered signaling can increase cellular oxidative stress. cAMP levels, adenylyl cyclase activity, and protein kinase A activity were measured using commercial kits. Generation of reactive oxygen species (ROS) was quantified using the fluorochrome dichlorofluorescein diacetate. Oxidative DNA damage was quantified using the single-cell electrophoresis technique. Here, we report that cells exposed to cell surface SE-positive HLA-DR (human leukocyte antigen-DR) molecules, to cell-free recombinant proteins genetically engineered to express the SE motif, or to SE-positive synthetic peptide showed diminished cAMP-dependent signaling, increased ROS levels, and higher vulnerability to oxidative DNA damage. Introduction of single amino acid substitutions into SE-positive peptides revealed a consensus five-amino acid sequence motif of Q/R-K/R-X-X-A that is necessary and sufficient for SE-triggered signaling. The pro-oxidative effect of the SE could be reversed by inhibiting NO production. We conclude that the SE acts as a signaling ligand that activates an NO-mediated pro-oxidative pathway. The potential contribution of this signaling aberration to RA pathogenesis is discussed.

## Introduction

The vast majority of patients with rheumatoid arthritis (RA) carry human leukocyte antigen-DRB1 (*HLA-DRB1*) alleles encoding QKRAA, QRRAA, or RRRAA amino acid sequences in positions 70 to 74 of the DR-β chain, a motif commonly known as the RA 'shared epitope' (SE) [1]. Immunogenetic analyses have identified the SE as the main genetic risk factor in diverse ethnic groups [2] and have documented a particularly strong association with a severe form of the disease [3]. It has also been suggested that a gene-dose effect may exist in which patients carrying two SE-positive alleles are afflicted with more severe disease than patients with one SE allele or none [4].

Despite the major role of genetic factors in RA susceptibility, the concordance of this disease in monozygotic twins is only 15% [5]. It has therefore been hypothesized that whereas RA susceptibility is determined genetically, disease onset may depend on stochastic non-genetic or epigenetic events. Indeed, increased incidence of stochastically occurring events such as protein and lipid damage, accelerated telomere shortening, DNA damage, and somatic mutations have been observed in RA patients [6-8] and in non-RA patients carrying the SE [9]. It is noteworthy that a common denominator for these events is their association with – or inducibility by – oxidative stress, which has long been implicated in RA [10-15]. Thus, a conceivable unifying explanation is that the SE increases susceptibility to RA by conducing to a pro-oxidative milieu, which increases the risk of deleterious stochastic events. Consistent with this model, a gene-environment interaction between the SE and smoking, a well-known pro-oxidant stressor [16], has been recently reported [17], with relative risks for RA of 7.5 in SE-positive smokers, 2.4 in smokers who were SE-negative, and 2.8 in SE-positive individuals without a history of smoking. That study also demonstrated a gene-dose effect, with a relative risk of 15.7 in smokers carrying two copies of the SE [17].

In this study, we investigated whether the SE plays a role in oxidative stress regulation. In prior studies [18,19], we identified two counteracting pathways that regulate cell sensitivity to oxidative challenges: an anti-oxidative pathway, which is triggered by extracellular adenosine and is mediated by cAMP-dependent protein kinase A (PKA), and a pro-oxidative, nitric oxide (NO)-mediated pathway that counteracts the cAMP-mediated pathway, thereby rendering cells more vulnerable to oxidative damage [18]. More recently, we demonstrated that the SE acts as a ligand that activates NO signaling in opposite cells in an allele-specific manner [20]. Here, we report that SE-triggered NO signaling blocks the cAMP-mediated anti-oxidative pathway and leads to increased vulnerability to oxidative damage. The SE effect is critically dependent on amino acids Q/R70, K/R71, and A74 of the DR-β chain, the very same residues previously shown to correlate best with RA susceptibility in population studies [21].

## Materials and methods

### Cells, HLA-DR typing, and culture conditions

B-lymphocyte lines prepared from peripheral blood by Epstein-Barr virus (EBV) transformation were grown in supplemented RPMI-1640 medium. B-cell lines were HLA-DR-typed using commercial, polymerase chain reaction-based, low-medium-resolution, DRB1 typing kits (Dynal Biotech, now part of Invitrogen Corporation, Carlsbad, CA, USA) followed by high-resolution, *DRB1 *allele-specific typing when indicated. A total of 40 donors were studied. Thirty-two of them carried one or two of the following SE-positive *HLA-DRB1 *alleles: *0101, *0401, *0404, *0405, or *1001. Eight cell lines carrying the *DRB1 *alleles *0301, *07, *08, *13, *15, or *11 composed the SE-negative group. SE-positive and SE-negative B-cell lines displayed equivalent levels of HLA-DR surface expression, as determined by flow cytometry analysis on 10 randomly selected lines (Additional file 1). The murine L-cell transfectants expressing human HLA-DR-α/β heterodimers were generated as described [22] and maintained in Dulbecco's modified Eagle's medium (DMEM) supplemented with 10 mM HEPES, 2 mM glutamine, 1% penicillin/streptomycin, 10% fetal calf serum, and 500 μg/ml G418. All L-cell transfectants displayed equivalent levels of HLA-DR surface expression, as determined by flow cytometry analysis (Additional file 1). Human M1 fibroblasts were grown in DMEM containing 10% fetal bovine serum, penicillin/streptomycin, and 10 mM HEPES buffer solution.

### Synthesis and solid-phase immobilization of peptides

Peptides were synthesized at the University of Michigan Protein Structure Facility on a Rainin PTI Symphony automated peptide synthesizer (Protein Technologies, Inc. Tucson AZ, USA). Each residue was coupled twice with 200 mM HOBt + HBTU and 400 mM *N*-methylmorpholine for 60 minutes and capped with 50% acetic anhydride in dimethylformamide. The resins used were Fmoc-PAL-PEG resins from Applied Biosystems (Foster City, CA, USA). Peptides were purified to more than 90% by high-performance liquid chromatography. All peptides were C- and N-terminally blocked. The sequences of the peptides used in this study and the *DRB1 *alleles that correspond to them are shown in Table 1.

To immobilize peptides on a solid phase, cyanogen bromide-activated Sepharose 4B beads were washed with 1 mM HCl and incubated with peptides in 0.1 M NaHCO_3 _and 0.5 M NaCl (pH 8.0) buffer overnight at 4°C. Five milligrams of peptide was mixed with 1 ml of Sepharose 4B beads. Free Sepharose groups were blocked with 0.2 M glycine (pH 8.0) for 2 hours at room temperature. Columns were washed at 4°C with the following buffers: 0.1 M NaHCO_3_, 0.5 M NaCl (pH 8.0) buffer, 0.5 M CH_3_COONa (pH 4.0) buffer, and finally phosphate-buffered saline (PBS) at pH 7.5. The efficiency of binding was determined by quantification of the residual concentration of peptides in the buffer after overnight incubation with the beads.

### SE-expressing recombinant proteins

HLA-DR tetramers DRB1*0401/DRA1*0101 (designated here as T-DRB1*0401) and DRB1*1501/DRA1*0101 (T-DRB1*1501) containing class II-associated invariant chain peptide (CLIP) in the antigenic groove were generated by the National Institutes of Health Tetramer Facility as previously described [23]. Chimeric hepatitis B core (HBc) particles expressing DR-β HV3 (the third allelic hypervariable region) were prepared as previously described [20].

### Membrane preparation

To prepare membranes, 5 × 10^5 ^cells were washed in cold PBS and resuspended in 300 μl of 10 mM Tris-HCl lysis buffer (pH 7.5) containing 3 mM dithiothreitol (DTT), 400 μM DGTA (diacylglycerylhydroxymethyltrimethyl-beta-alanine), 2 mM MgCI_2_, 0.1 mg/ml PMSF (phenylmethylsulfonyl fluoride), and 1 μg/ml each pepstatin and leupeptine. Cells were homogenized using a glass Dounce Tissue Grinder (Wheaton Science Products, Millville, NJ, USA). The homogenates were centrifuged at 1,000 *g *for 10 minutes at 4°C. The supernatants were then centrifuged at 12,000 *g *for 30 minutes. The resulting particulate fractions were suspended in 20 mM Tris-HCl buffer (pH 7.5) containing 60 mM KCl and 1 mM ethylenediaminetetraacetic acid (EDTA). Protein concentration was determined by the Lowry method.

### Measurement of cAMP

Experiments were performed in a serum-free RPMI-1640 medium containing 1% Nutridoma HU (Boehringer Mannheim, now part of Roche Diagnostics, Basel, Switzerland), 10 mM HEPES, 200 mM glutamine, and 1% penicillin/streptomycin. Cells were stimulated with 25 μM forskolin for the indicated time. The stimulation was stopped by adding one volume of 10% trichloroacetic acid, followed by sonication. The lysates were centrifuged at 12,000 *g *for 15 minutes at 4°C, and the supernatants were extracted three times with five volumes of diethyl ether. The residual ether was removed by heating the samples to 70°C for 15 minutes. A kit from Cayman Chemical Company (Ann Arbor, MI, USA) was used to measure cAMP.

### Measurement of PKA activity

By means of a commercial kit (Gibco-BRL, now part of Invitrogen Corporation, Carlsbad, CA, USA), PKA activity was quantified based on the transfer of phosphate-32 from γ-P^32 ^ATP to the synthetic heptapeptide Leu-Arg-Arg-Ala-Ser-Leu-Gly. Results are expressed as the percentage of activity relative to maximal activity by a positive control provided with the kit.

### Adenylyl cyclase activity assay

Cell membranes were prepared as described above. The composition of the reaction buffer was 50 mM triethanolamine (pH 7.4), 1 mM EDTA, 1 mM DTT, 5 mM MgCI_2_, 5 mM creatine phosphate, 20 mM rolipram, 1 mM IBMX (3-isobutyl-1-methylxanthine), 1 mM ATP, 0.05 mM guanosine triphosphate, 60 U/ml creatine phosphate kinase, 4 units per milliliter each of myokinase and adenosine deaminase type VIII (Sigma-Aldrich, St. Louis, MO, USA), 60 mM KCl, and 0.2% bovine serum albumin. The reaction was initiated by the sequential addition of the cell membrane suspension (10 μg of proteins) to the incubation medium. The total reaction volume was 70 μl. Incubations were performed for 5 minutes at 32°C. To stop the reaction, 2 μl of 0.5 M EDTA (pH 8.0) was added, followed by boiling for 10 minutes. cAMP generation in the reaction, representing adenylyl cyclase (AC) activity, was determined using a cAMP assay kit (Amersham, now part of GE Healthcare, Little Chalfont, Buckinghamshire, UK).

### Measurement of reactive oxygen species levels

To measure spontaneous reactive oxygen species (ROS) production by B lymphocytes, cells were first loaded with 10 μM 2',7'-dichlorodihydrofluorescein (DCFH-DA) for 30 minutes, washed, and plated at a density of 1 × 10^5 ^per well in round-bottom 96-well plates (OptiPlate™ 96F; PerkinElmer Life And Analytical Sciences, Inc., Waltham, MA, USA) in 100 μl of DMEM-phenol red-free medium. Fluorescence level was recorded continuously over a period of 200 minutes by a Fusion™ αHT system (PerkinElmer Life And Analytical Sciences, Inc.) at an excitation wavelength of 485 nm and an emission wavelength of 530 nm.

To measure ROS in adherent M1 cells, sub-confluent cultures were detached from flasks using 0.05% trypsin, and 5 × 10^3 ^cells per well were seeded into 96-well plates. After an overnight culture, cells were washed with DMEM-phenol red-free medium twice and loaded with DCFH-DA, and dichlorofluorescein (DCF) fluorescence was recorded as above.

### Quantification of oxidative DNA damage by the comet assay

Sub-confluent fibroblasts were detached from the tissue-culture flasks with 0.05% trypsin solution. After centrifugation, cells were washed and re-suspended in serum-free DMEM, divided into 1.5-ml Eppendorf tubes, and centrifuged at 2,500 rpm for 10 minutes. Ten micromolar 2-chloroadenosine (2CA) was added and samples were kept in a 37°C incubator for 30 minutes. In some experiments, cells were pre-incubated with peptides or various inhibitors for the indicated times at 37°C prior to exposure to 2CA. At the end of incubation, cells were washed with PBS and treated with 100 μM H_2_O_2 _in 0.5 ml of PBS on ice for 20 minutes. At the end of H_2_O_2 _treatment, cells were immediately microfuged for 15 seconds. The cells were washed with cold PBS twice and re-suspended at 1 × 10^5 ^cells per milliliter in ice-cold PBS. Five microliters of cell suspension was mixed with 50 μl of molten LMAgarose, and the mixture was placed onto CometSlide™ (Trevigen, Inc., Gaithersburg, MD, USA). Slides were placed at 4°C in the dark for 10 minutes, immersed in pre-chilled lysis solution (Trevigen, Inc.), and kept at 4°C for an additional 30 minutes. At the end of incubation, the slides were placed in a 50-ml Coplin jar containing alkali buffer for 30 minutes at room temperature in the dark and then immersed in 50 ml of Tris borate-EDTA (TBE) buffer for 5 minutes. Slides were then placed flat onto a gel tray submerged in TBE buffer in a horizontal electrophoresis apparatus and were aligned equidistant from the electrodes. Electrophoresis was performed at 1 V/cm for 10 minutes. At the end of electrophoresis, samples were fixed in 100% methanol for 5 minutes and 100% ethanol for 5 minutes. After drying, 50 μl of diluted SYBR Green was added onto each circle of agarose. Comet tail fluorescence intensity was quantified with a Diagnostic Instruments (Sterling Heights, MI, USA) digitized camera mounted on a Nikon Eclipse E400 (Nikon USA, Melville, NY, USA) microscope, using Scion Image software (Scion Corporation, Frederick, MD, USA). Quantification of comet fluorescence was performed as previously described [18]. Each data point was derived from 30 to 100 individual cells. To mitigate unavoidable inter-experimental variability in fluorescent units and to allow better comparisons among different experiments, the data are shown as the percentage of DNA damage protection (the percentage of decrement in tail fluorescence intensity with 2CA relative to the intensity recorded in cells treated with H_2_O_2 _only) ± standard error of the mean. As an illustrative example, in medium, M1 cells had a background of 258,791 ± 27,930 fluorescence units (A). Exposure of these cells to H_2_O_2 _resulted in 1,549,668 ± 163,699 units (B) but in the presence of 2CA was only 885,197 ± 86,147 units (C). The calculated DNA damage protection by 2CA in medium was therefore 51.47% ± 6.3% (1 - [(C - A)/(B - A)] × 100). In contrast, in the presence of an SE-expressing ligand, the corresponding counts were 217,921 ± 25,483 units (A), 1,408,434 ± 179,542 units (B), and 1,442,636 ± 162,897 units (C), with a calculated DNA damage protection rate of -2.87% ± 8.89%.

### Statistical analysis

Unless stated otherwise, Student's *t *test was used. Statistical significance was achieved at a *p *value of less than 0.05 (marked with an asterisk). Dose-response curves and calculation of half inhibitory concentration (*IC*_50_) were performed using PRISM 3.0 software (GraphPad Software, Inc., San Diego, CA, USA).

## Results

**Figure 1:**
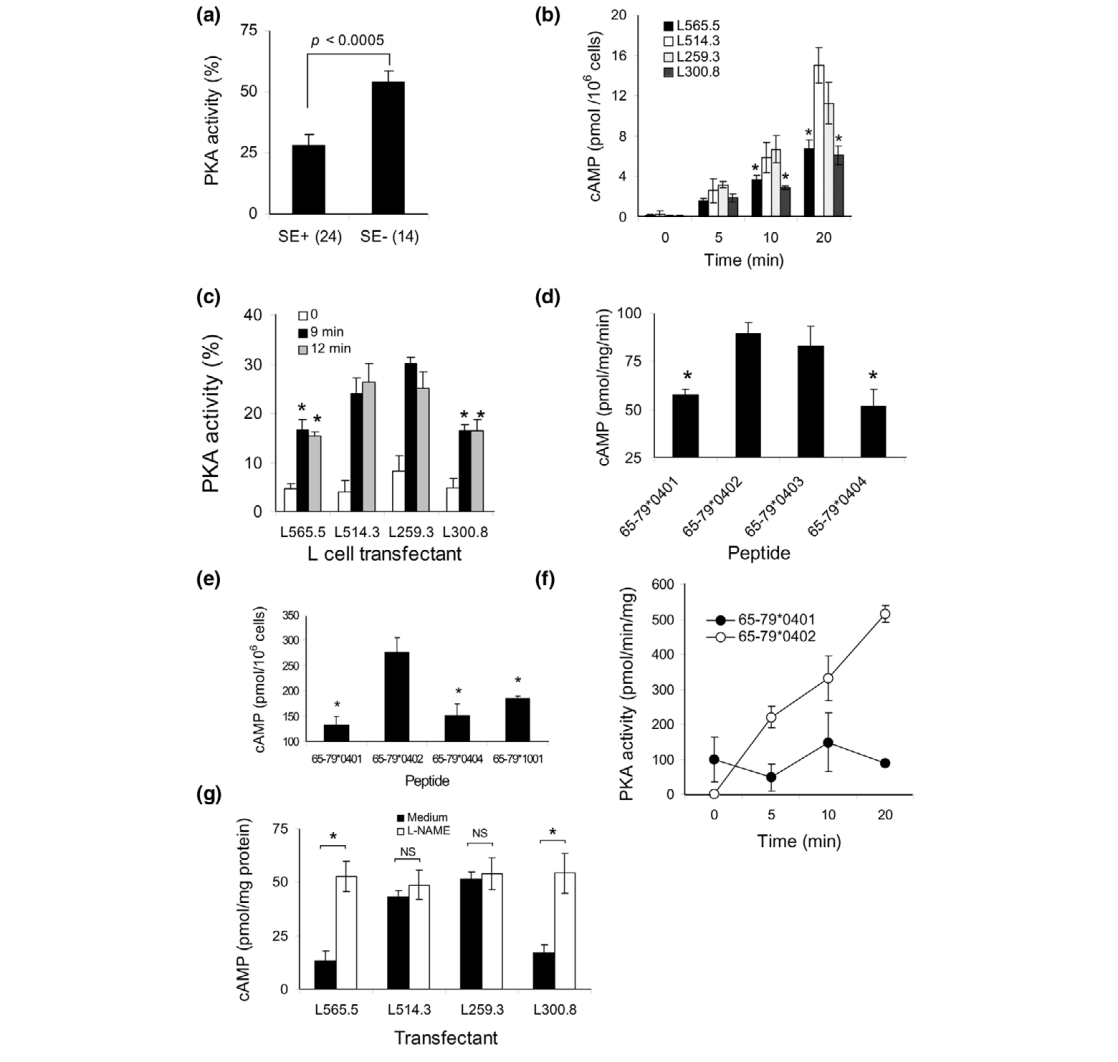
Inhibition of cAMP-dependent signaling by the shared epitope (SE). **(a) **Epstein-Barr virus-transformed B-cell lines expressing SE-positive or SE-negative human leukocyte antigen-DRB1 (*HLA-DRB1*) alleles were stimulated with the adenylyl cyclase (AC) activator forskolin (25 μM) for 12 minutes, and protein kinase A (PKA) activity was determined. The values in parentheses represent the number of donors in each group. **(b) **L-cell transfectants expressing SE-positive or SE-negative *HLA-DRB1 *alleles were stimulated with the AC activator forskolin (25 μM) over a time course, and cAMP levels were quantified. **(c) **L-cell transfectants were stimulated with forskolin as in **(b)**, and PKA activity was determined. **(d) **M1 cells were pre-incubated overnight with 50 μg/ml of different soluble 15 mer synthetic peptides corresponding to the HV3 region (residues 65 to 79) encoded by SE-positive or SE-negative *HLA-DRB1 *alleles. Cells were then stimulated with 25 μM forskolin, and cell membrane AC activity was determined. **(e) **Intact M1 cells were pre-incubated overnight with either SE-positive or SE-negative soluble 15 mer peptides, and cAMP levels were determined 12 minutes after stimulation with forskolin. **(f) **M1 cells were pre-incubated for 1 hour with different 15 mer peptides immobilized on Sepharose beads and then stimulated with 2-chloroadenosine (2CA, 1 μM), and PKA activity was measured over a time course. Black circles indicate SE-positive peptide 65–79*0401, and white circles indicate SE-negative peptide 65–79*0402. **(g) **L-cell transfectants expressing SE-positive or SE-negative *HLA-DRB1 *alleles were pre-incubated for 15 minutes in the presence or absence of the non-selective nitric oxide synthase inhibitor *N*-nitro-L-arginine methyl ester (L-NAME, 4 mM) and then stimulated with forskolin (25 μM), and cAMP levels were measured. NS, not significant.

We have recently demonstrated that the SE acts as a ligand capable of triggering NO signaling in opposite cells [20]. Consistent with the known inhibitory effect of NO on the cAMP-mediated pathway [24,25], cells encountering the SE displayed impaired cAMP signaling (Figure 1). For example, EBV-transformed B-cell lines expressing SE-encoding *HLA-DRB1 *alleles had a much lower PKA activation in response to stimulation with the AC-stimulating agent forskolin compared to SE-negative lines (Figure 1a). To determine whether the signaling aberration was attributable to the *DRB1 *gene itself or was a result of another gene secondary to linkage disequilibrium, we studied murine L-cell transfectants expressing human HLA-DR-α/β heterodimers on their surface [22]. As can be seen in Figure 1b, L cells expressing SE-positive DR-β chains on their surface through cDNA transfection (lines L565.5 and L300.8, expressing alleles DRB1*0401 and DRB1*0404, respectively) produced significantly less cAMP in response to forskolin compared to transfectants expressing SE-negative DR-β chains (lines L514.3 and L259.3, expressing alleles DRB1*0402 and DRB1*0403, respectively). Activation of the cAMP-dependent kinase PKA was likewise significantly lower in SE-expressing transfectants compared to transfectant lines expressing SE-negative alleles (Figure 1c). We have verified that the observed findings are not a transfection-associated artifact in which a seemingly lower cAMP activation in SE-expressing transfectants is a mere reflection of an enhancing effect of the SE-negative transfected genes rather than true inhibition by the SE-positive genes. To address this question, we compared cAMP signaling between SE-expressing transfectants and untransfected cells or cells transfected with a vector that does not allow surface expression of the DR molecule (Additional file 2). In both cases, SE-expressing transfectants displayed markedly lower cAMP activation. Thus, the SE effect was not due to an artifact caused by transfection but represented a *bona fide *inhibition of cAMP signaling.

Consistent with our previous data [20], synthetic 15 mer peptides containing SE sequences could mimic the SE effect in its natural conformation. As can be seen, such peptides inhibited forskolin-induced AC activation (Figure 1d) and generation of cAMP (Figure 1e), resulting in inhibition of PKA activation (Figure 1f). The inhibitory effect of the SE was mediated by NO. As shown in Figure 1g, L-cell transfectants expressing SE-positive HLA-DR molecules had impaired cAMP response to activation by forskolin. However, this impairment was completely reversed in cells that had been pre-incubated with the NO synthase (NOS) inhibitor L-NAME (*N*-nitro-L-arginine) (Figure 1g). Taken together, these data indicate that the SE inhibits cAMP-mediated signaling due to its activation of an antagonistic, NO-mediated pathway.

**Figure 2:**
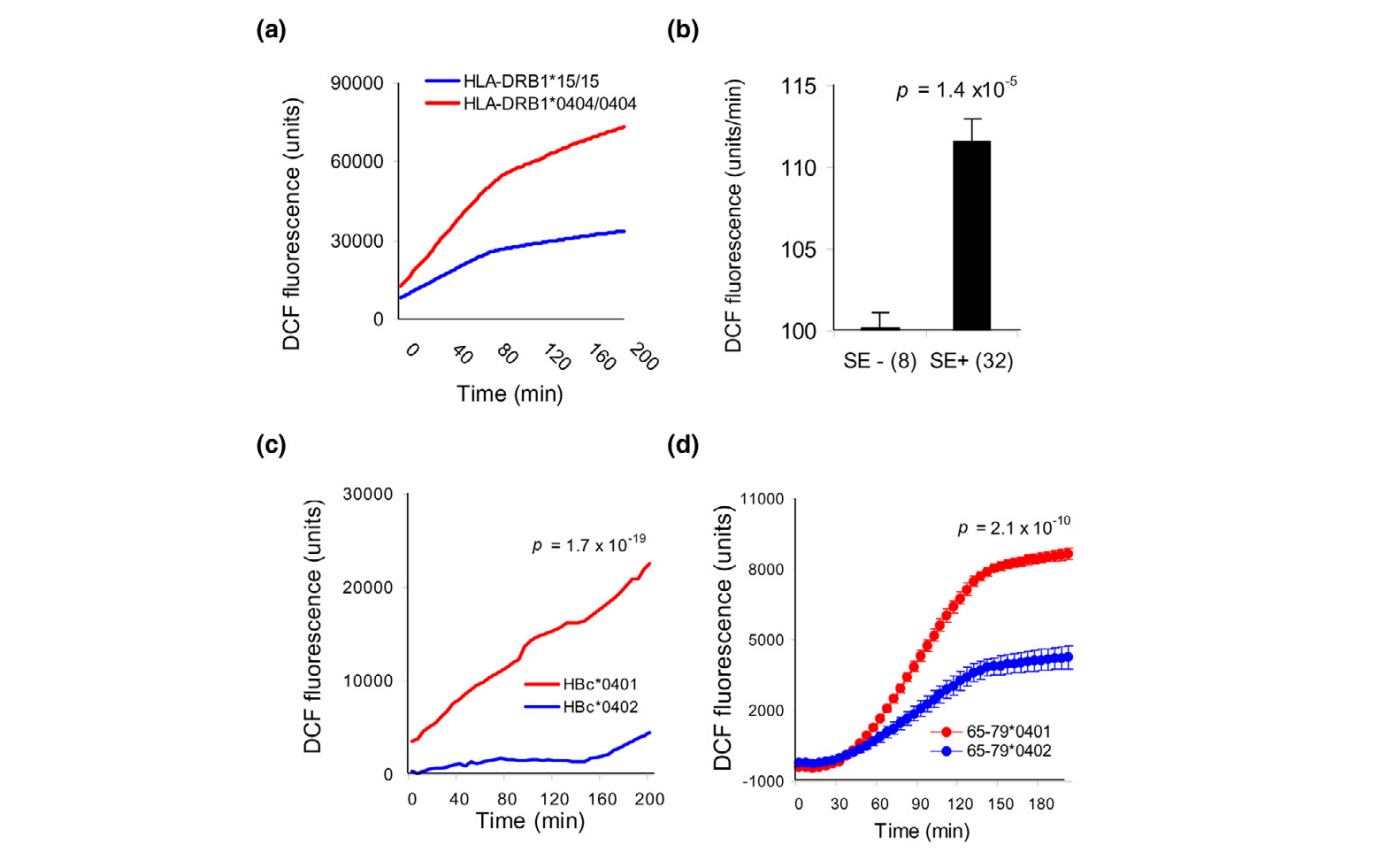
The shared epitope (SE) accelerates endogenous reactive oxygen species (ROS) production. **(a) **Time-course ROS levels in representative SE-positive (red line) and SE-negative (blue line) B-lymphocyte cell lines. **(b) **ROS production rates in B lymphocytes. Results are shown as the mean ± standard error of the mean fluorescent units per minute (FU/minute) during the linear late phase of the ROS curve (80 to 200 minutes). Data of the SE-positive (SE+) and SE-negative (SE-) groups were compiled from three to five consecutive experiments in each cell line. The values in parentheses represent the number of donors in each group. **(c) **ROS levels in M1 cells stimulated with 2.5 μg/ml of either SE-positive hepatitis B core (HBc) capsids (HBc*0401, red line) or SE-negative HBc capsids (HBc*0402, blue line). **(d) **ROS levels in M1 cells stimulated with 100 μg/ml of either an SE-positive 15 mer peptide (65–79*0401, red circles) or an SE-negative 15 mer peptide (65–79*0402, blue circles). Calculations of *p *values in **(c) **and **(d) **were based on paired *t *test analysis. DCF, dichlorofluorescein.

The cAMP-mediated pathway has been previously shown to activate an anti-oxidative cellular response, whereas NO has been found to exert a pro-oxidative effect [18]. Given the diametrically opposite effects of the SE on the cAMP- and the NO-mediated pathways on the one hand and the proposed role of oxidative stress in the pathogenesis of RA [10-15] on the other, we proceeded to examine whether the SE could affect ROS production. To that end, we first studied spontaneous ROS levels in B-lymphocyte lines from 40 HLA-DR-typed individuals. Cells were loaded with the fluorescent ROS probe DCFH-DA and were cultured over a time course at high density to allow close cell-cell contact. As shown in Figure 2, SE-positive cells demonstrated a robust spontaneous ROS production. Curves of two representative cell lines are shown to illustrate the difference between SE-positive and SE-negative cells (Figure 2a). It is noteworthy that the ROS curves showed a biphasic shape with a steeper slope during the first hour. The reason for this pattern is unknown; however, it could represent an additional mechanical stress in the early phase, during which cells are adapting to the high-density culture conditions. To avoid this potential artifact, we based the calculation of ROS production rates on the later phase of the curve (80 to 200 minutes). As can be seen in Figure 2b, mean ROS production rates were markedly higher in the SE-positive group compared to the SE-negative group (*p *= 1.4 × 10^-5^).

To investigate whether the SE is directly involved, we used recombinant multimeric HBc molecules engineered to express HV3 (residues 65 to 79) of different *DRB1*-encoded DR-β chains [20]. HBc multimeric proteins are efficient non-replicative and non-infective carriers of foreign epitopes [26]. Importantly, the tips of each spike form α-helical structures, mimicking the native conformation of the HV3. Oligonucleotides encoding residues 65 to 79 of either the SE-positive allele DRB1*0401 or the SE-negative allele DRB1*0402 were inserted at the tips of the HBc spikes as we previously described [20]. As can be seen in Figure 2c, HLA class II-negative M1 cells produced much higher ROS levels when incubated with the SE-expressing multimeric protein HBc*0401 compared to cells incubated with the SE-negative control protein HBc*0402. Similarly, M1 cells incubated with the SE-positive 15 mer peptide 65–79*0401 produced a much more robust ROS response compared to cells incubated with the control 15 mer peptide 65–79*0402 (Figure 2d). Thus, accelerated ROS production can be seen in cells upon contact with other SE-expressing cells or with cell-free SE ligands.

**Figure 3:**
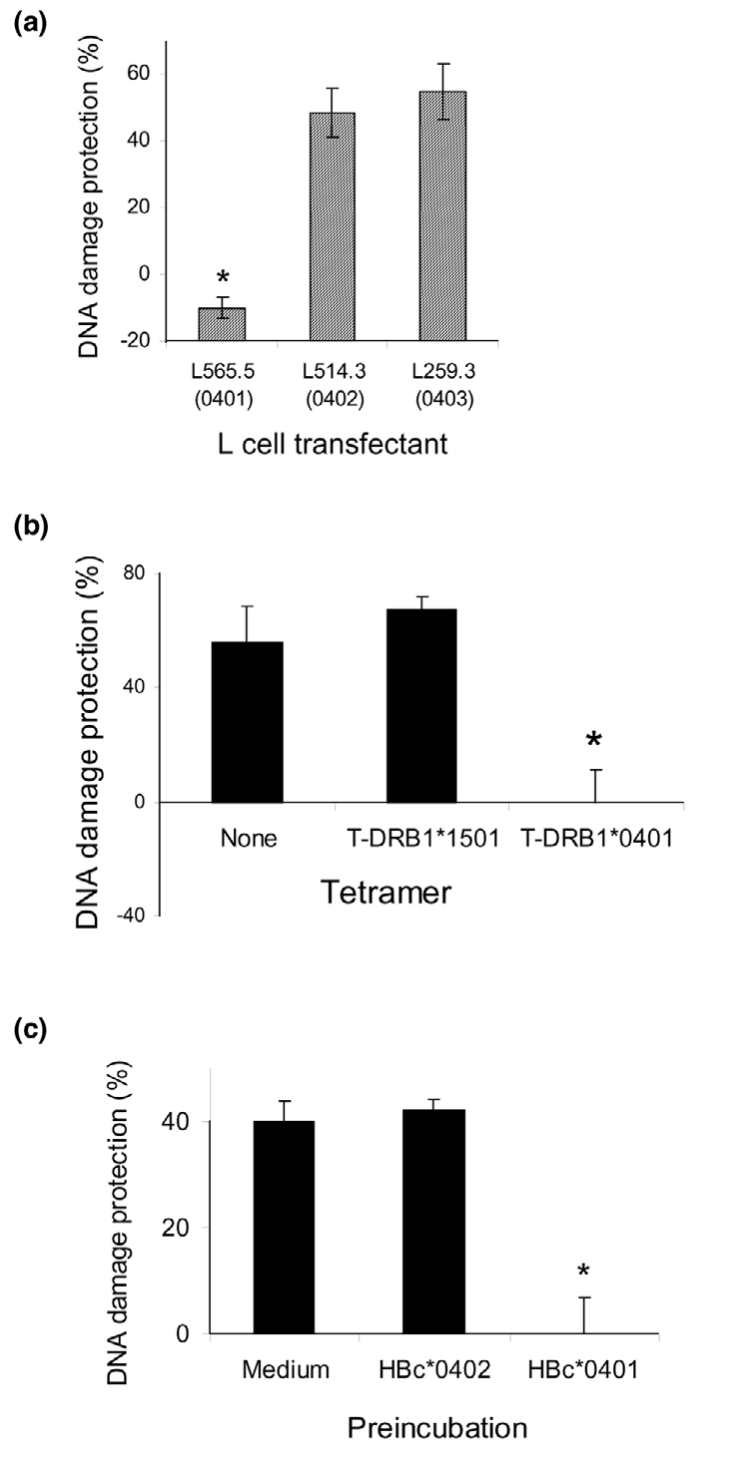
The shared epitope (SE) attenuates adenosine-induced protection against oxidative DNA damage. **(a) **The potency of adenosine-mediated anti-oxidative signaling was determined in L-cell transfectants expressing cDNA corresponding to DRB1*0401 (L565.5), DRB1*0402 (L514.3), or DRB1*0403 (L259.3). **(b) **Human M1 fibroblasts were incubated for 1 hour with or without 24 μg/ml of SE-positive (T-DRB1*0401) or SE-negative (T-DRB1*0401) tetrameric molecules. At the end of incubation, the extent of adenosine-induced protection against oxidative DNA damage was determined as above. **(c) **M1 human fibroblasts were pre-incubated overnight in the absence or presence of 2.5 μg/ml of either HBc*0401 or HBc*0402 multimeric proteins. At the end of incubation, the extent of adenosine-induced protection against oxidative DNA damage was determined. *p < 0.05.

Among its various functions, NO has been found to increase susceptibility to oxidative DNA damage by counteracting the genoprotective effect of the adenosine-activated cAMP pathway [18]. Given our previous findings that the SE can trigger NO production [20], which in turn inhibits cAMP signaling (Figure 1), we proceeded to determine whether cells carrying SE-encoding *DRB1 *alleles are more susceptible to DNA damage. To that end, adenosine-treated L-cell transfectants were challenged with H_2_O_2 _and the extent of DNA damage was quantified. As can be seen in Figure 3a, SE-negative transfectants L514.3 and L259.3 mounted adequate adenosine-mediated resistance to H_2_O_2_-induced DNA damage, whereas SE-positive transfectant L565.5 cells failed to respond to adenosine and consequently suffered more extensive DNA damage.

To investigate whether the DR molecule is directly involved, we used SE-positive and SE-negative HLA-DR tetramers. The assembly of major histocompatibility complex (MHC) class II tetrameric molecules depends on the presence of an antigenic groove peptide that should conform to a sequence motif, which is distinct for each allele. To allow comparisons between the SE-negative and SE-positive tetramers independent of allele-specific groove peptides, we used tetramers containing a covalently bound CLIP [23]. As can be seen in Figure 3b, adenosine-treated M1 fibroblasts stimulated with the SE-positive T-DRB1*0401 tetramer sustained much more DNA damage when challenged with H_2_O_2 _compared to cells incubated with the control, SE-negative T-DRB1*1501 tetramer. Because T-DRB1*0401 and T-DRB1*1501 share identical DR-α chains and groove peptides, their differential signaling activity can be attributed only to their distinct DR-β chains. Inhibition of adenosine-mediated resistance to oxidative DNA damage could also be seen in cells incubated with the SE-expressing multimeric protein HBc*0401 as compared to cells incubated with the SE-negative capsids HBc*0402 (Figure 3c).

**Figure 4:**
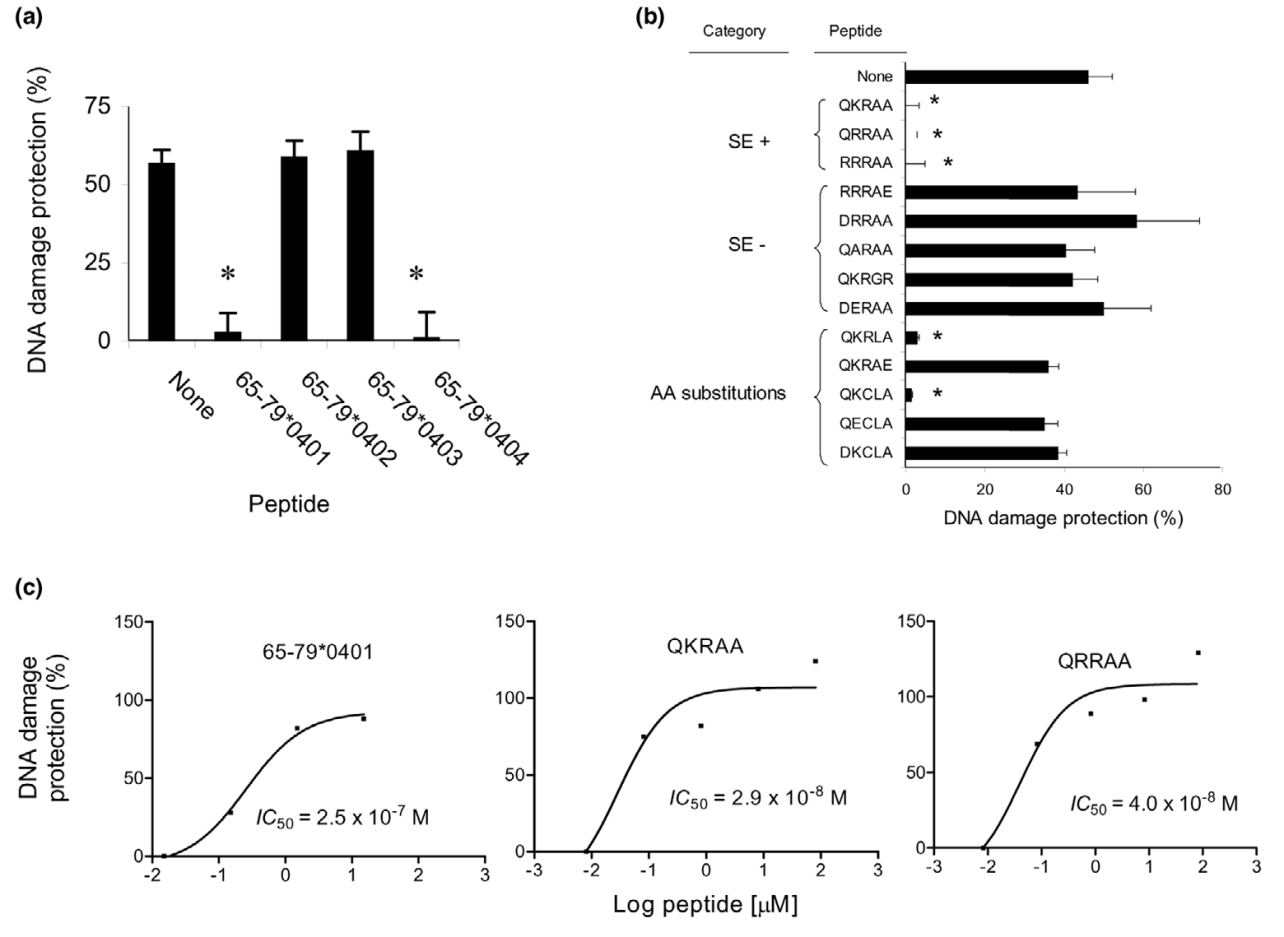
Pro-oxidative signaling by shared epitope (SE)-expressing peptides. **(a,b) **The potency of adenosine-induced anti-oxidative signaling was determined in human M1 fibroblasts, which had been pre-incubated overnight with 50 μg/ml of different 15 mer **(a) **or 5 mer **(b) **peptides. *p < 0.05. **(c) **Dose-response analysis of soluble peptide-mediated pro-oxidative effect. AA, amino acid; *IC*_50_, half inhibitory concentration.

Pro-oxidative signaling could also be triggered by synthetic peptides containing the SE sequence (Figure 4). As can be seen, cells incubated with SE-positive 15 mer peptides 65–79*0401 or 65–79*0404 prior to treatment with adenosine and then exposed to H_2_O_2 _suffered more oxidative DNA damage. Control peptides derived from the SE-negative alleles DRB1*0402 (designated 65–79*0402) or DRB1*0403 (designated 65–79*0403) had no effect (Figure 4a).

Peptides 65–79*0404 and 65–79*0403 differ by a single amino acid residue (A74 versus E74, respectively) (Table 1). This maps the active region to the SE itself (residues 70 to 74). To ascertain this possibility, we synthesized 5 mer peptides corresponding to the SE (QKRAA, QRRAA, and RRRAA, representing alleles DRB1*0401, DRB1*0404, and DRB1*1001, respectively). As can be seen in Figure 4b, 5 mer peptides QKRAA, QRRAA, and RRRAA all had a pro-oxidative effect, whereas 5 mer peptides expressing non-SE sequences (for example, RRRAE, DRRAA, QKRGR, or DERAA) had no such effect. To determine which of the five SE amino acid residues are critical, we introduced single amino acid substitutions into the 5 mer peptide QKRAA. We found that Q/R70, K/R71, and A74 are critical amino acids for the SE effect, whereas residues 72 and 73 do not seem to play a role (Figure 4b). Thus, a consensus motif Q/R-K/R-X-X-A was identified as necessary and sufficient for SE signaling. Synthetic peptides showed effectiveness at nanomolar concentrations. The *IC*_50 _values of their pro-oxidative effects were 250 nM for peptide 65–79*0401, 29 nM for peptide QKRAA, and 40 nM for QRRAA (Figure 4c).

**Figure 5:**
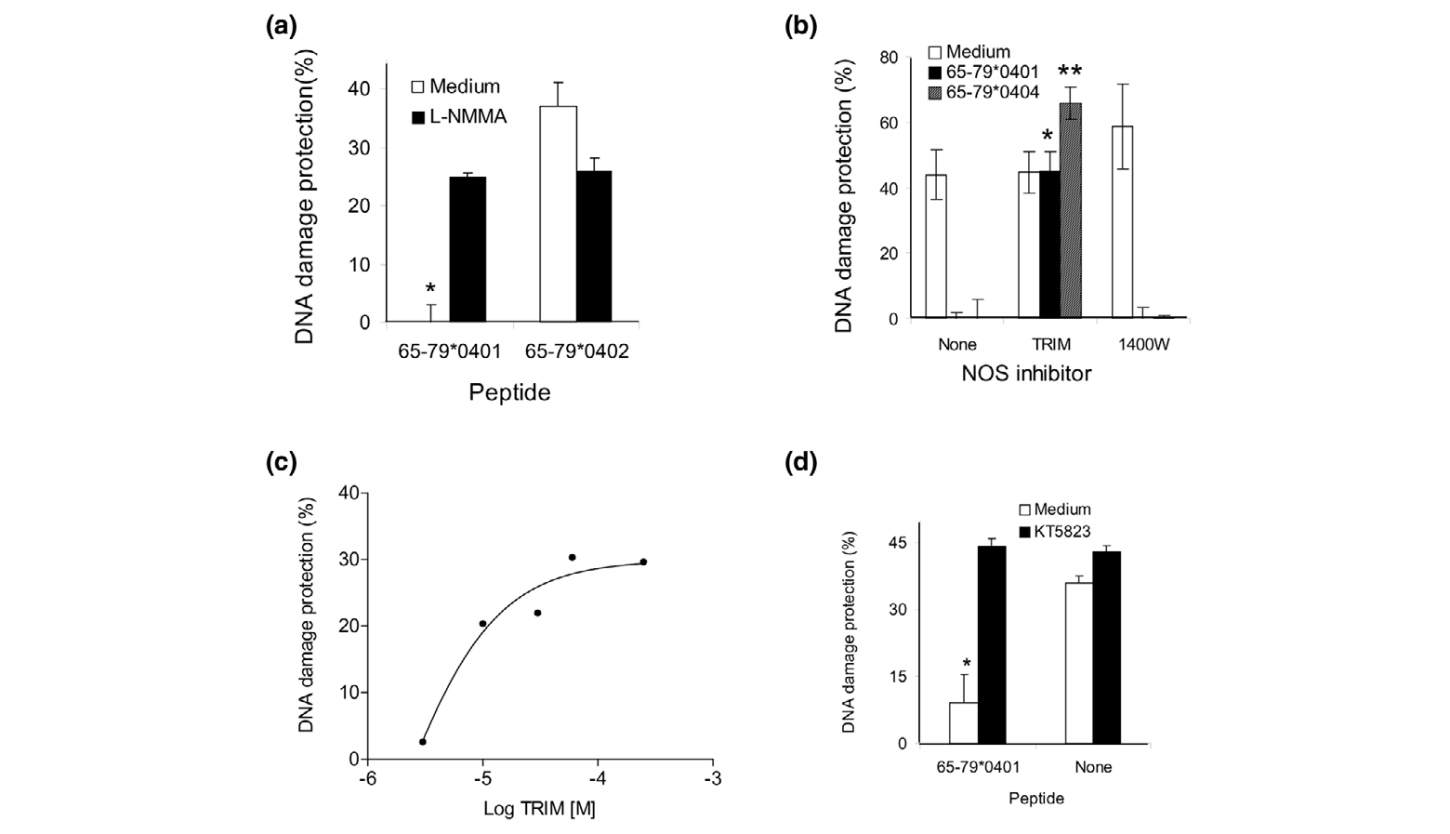
Mapping the shared epitope (SE)-triggered pro-oxidative signaling pathway. **(a) **M1 cells were pre-incubated for 20 minutes with the nitric oxide synthase (NOS) inhibitor L-NMMA (500 μM) and exposed to Sepharose bead-immobilized allele-specific 15 mer peptides for 1 hour, and the extent of adenosine-induced protection against oxidative DNA damage was determined. **(b) **M1 cells were incubated with or without 50 μg/ml of peptide 65–79*0401 or 65–79*0404 in the presence or absence of NOS1 inhibitor TRIM (trifluoromethylphenylimidazole) (250 μM) or the NOS2-selective inhibitor 1400 W (0.2 μM). After overnight incubation, cells were washed, stimulated with 2-chloroadenosine, followed by oxidative challenge with H_2_O_2_, and the extent of DNA damage was quantified. The concentration of each inhibitor was adjusted to 10-fold higher than its *IC*_50_. *p <0.02; **p <10^-5^. **(c) **Dose-response curve of TRIM effect. Cells were incubated overnight with 50 μg/ml of peptide 65–79*0404 in the presence of different concentrations of TRIM. At the end of incubation, cells were collected and adenosine-induced protection against DNA damage was quantified as above. **(d) **M1 cells were pre-incubated for 10 minutes with the PKG inhibitor KT5823 (1 μM) and subsequently exposed to Sepharose bead-immobilized peptide 65–79*0401. The extent of adenosine-induced protection against oxidative DNA damage was determined. *IC*_50_, half inhibitory concentration; L-NMMA, L-*N*^G^-monomethyl arginine citrate; PKG, cGMP-dependent kinase.

Finally, the involvement of NO in SE-triggered pro-oxidative signaling was investigated. As can be seen in Figure 5a, pre-incubation of M1 cells with the NOS inhibitor L-NMMA (L-*N*^G^-monomethyl arginine citrate) prevented the pro-oxidative effect of SE-expressing peptide 65–79*0401. The NOS1-selective inhibitor TRIM (trifluoromethylphenylimidazole), but not the potent NOS2-selective inhibitor 1400 W, reversed the SE-triggered pro-oxidative effect (Figure 5b,c). The PKG (cGMP-dependent kinase) inhibitor KT5823 reversed SE-triggered pro-oxidative effects, implicating this kinase as a likely downstream signaling molecule in the SE-triggered pro-oxidative pathway.

## Discussion

We report herein that the SE acts as an allele-specific ligand that inhibits an adenosine-mediated anti-oxidative pathway in opposite cells, thereby increasing cell vulnerability to oxidative damage. Both adenosine and the cAMP-mediated pathway have been previously shown to attenuate oxidative stresses [27-36]. Adenosine is a known anti-inflammatory and cytoprotective molecule secreted by injured cells in order to protect the bystander tissue [37,38]. For example, in cardiomyocytes, adenosine-mediated signaling has been found to attenuate oxidative stress during reoxygenation [34], and in mesangial cells, adenosine has been shown to inhibit phorbol myristate acetate-induced intracellular production of ROS [35]. We have recently demonstrated that activation of the cAMP pathway by extracellular adenosine via adenosine receptors of the A_2 _type protects cells against oxidative DNA damage. Increased NO levels, on the other hand, inhibit the protective effect of cAMP, thereby rendering cells more vulnerable to oxidative damage [18]. Thus, a balance between two pathways, mediated respectively by NO and cAMP, determines the extent of oxidative damage in cells.

Here, we demonstrate that the SE, when expressed in its native conformation on the cell surface, inhibits cAMP-dependent signaling. Synthetic peptides (15 mer) corresponding to the HV3 region of SE-positive HLA-DR molecules likewise inhibit the pathway in HLA class-II negative cells. By using NOS inhibitors, we further demonstrate that the effect of SE on cAMP-mediated signaling is due to its action as a ligand capable of activating NO-mediated signaling in opposite cells [20].

The molecular targets affected by the SE-triggered NO pathway are unknown. In other systems, NO has been shown to inhibit cAMP signaling either through a direct effect on AC or indirectly via activation of soluble guanylyl cyclase, resulting in cGMP-mediated activation of phosphodiesterases [24,25]. Inhibition of G_s _protein-coupled receptor activation is yet another potential mechanism [39]. The adenosine receptor, however, is a less likely target in the system described here, given the SE-inhibited activation by forskolin, which is a post-receptor event. Thus, the SE inhibitory effect is likely targeted at the AC level or further downstream.

Our experiments using isozyme-selective NOS inhibitors suggest that the mechanism of NO increase by SE involves activation of NOS1 (Figure 5) [20]. It should be cautioned, however, that the fidelity of chemical NOS inhibitors is imperfect. Additionally, the studies reported here involved fibroblasts. Whether a different NOS isozyme may mediate SE signaling in other cell types is unknown. Further studies using more specific inhibition techniques (for example, RNA interference) will be required to conclusively identify the NOS isozymes involved in different lineages.

We demonstrate here that, as a result of SE-triggered NO production, extracellular adenosine fails to activate the cAMP-mediated anti-oxidative pathway. Our results (Figure 2) further indicate that cells encountering the SE produce more ROS in the absence of any exogenously added oxidative challenge. Thus, the SE impairs the ability of cells to effectively use the cAMP-mediated anti-oxidative response. Because ROS mediate important intracellular signaling events [10,11], it is conceivable that SE-associated accumulation of ROS could have functional cell activation consequences. At excessive levels, ROS can also cause oxidative damage to macromolecules. It is noteworthy, therefore, that the extent of baseline DNA damage in unchallenged cells was not different in the presence or absence of the SE (data not shown). However, when exposed to exogenous oxidative challenges (that is, addition of H_2_O_2_), SE-encountering cells sustained more DNA damage than cells that had not encountered that ligand. Thus, these *in vitro *results suggest that, although the SE does not cause spontaneous oxidative damage, it could increase the risk of such damage during exogenous oxidative challenges. The *in vivo *implications of these findings are unknown, but it is tempting to speculate that in the absence of exogenous oxidative stress, individuals carrying SE-positive *HLA-DRB1 *alleles may experience only minor signaling aberrations resulting from the known immunomodulatory effects of ROS [10,11]. However, when challenged with exogenous oxidative stresses, these individuals may be at a higher risk of oxidative damage to macromolecules.

By using synthetic peptides corresponding to the HV3 region encoded by different *DRB1 *alleles (Figures 1 and 4), we demonstrate that the three known SE sequences (that is, QKRAA, QRRAA, and RRRAA) can all trigger pro-oxidative signals. We further demonstrate that an amino acid sequence motif of Q/R-K/R-X-X-A is necessary and sufficient for activation of the SE-triggered pathway. It is intriguing that the very same motif has been previously shown to correlate best with RA susceptibility in RA population studies [21].

We propose that these findings could provide new insights into some unresolved questions about the role of the SE in RA. The prevailing hypotheses to explain the association of SE and RA are based on the known role of HLA-DR molecules in antigen presentation. However, published data to support presentation of a specific antigen as a primary event in RA are equivocal. Moreover, the SE effect is not exclusive to RA; several other human diseases, as well as several animal models, have also been shown to be associated with SE-expressing *HLA-DRB1 *alleles [40-44]. One example of the enigmatic role of the SE is illustrated by transgenic mice expressing the SE-positive allele DRB1*0401. These mice have been found to develop a more severe experimental encephalomyelitis [45], a disease model that is induced by immunization against nervous system-derived antigens, which have little to do with arthritis. Thus, the SE demonstrates promiscuous association with pathogenically diverse diseases but with no apparent antigen- or species-specificity. It is therefore conceivable that, in addition to its role in the adaptive immune system, the SE possesses antigen-non-specific functions by activation of the innate immune system.

This study describes a novel innate immunity function of the SE: activation of a pro-oxidative NO-mediated signaling cascade. The pathway is activated within 1 hour after exposure to soluble peptides but can be triggered within minutes after stimulation with solid phase-immobilized peptides, suggesting that cross-linking of a cell surface receptor may be involved. The identity of the receptor is under study.

ROS and NO have been previously implicated as important players in the immune aberrations observed in RA. For example, increased ROS levels have been directly blamed for T-cell hyporesponsiveness [10,11], and NO-mediated resistance to Fas-induced apoptosis has been described in RA [14]. The data reported here offer a testable hypothesis regarding the role of SE in RA pathogenesis. Moreover, due to increased surface expression of class II MHC molecules in inflammatory sites, SE-triggered pro-oxidative effects offer a plausible explanation for the self-perpetuating nature of the disease. In addition, the cumulative effect of intercurrent exogenous oxidative challenges throughout life could conceivably render patients with RA more vulnerable to mutations [7,8] and premature telomeric loss [9].

## Conclusion

We demonstrate here that the SE acts as an allele-specific ligand that activates an NO-mediated pro-oxidative signaling in opposite cells. Based on the proposed role of oxidative stress in RA pathogenesis, it is tempting to speculate that the signaling effect reported here may contribute to disease susceptibility and/or severity in RA.

## Abbreviations

2CA = 2-chloroadenosine; AC = adenylyl cyclase; CLIP = class II-associated invariant chain peptide; DCFH-DA = 2',7'-dichlorodihydrofluorescein; DMEM = Dulbecco's modified Eagle's medium; DTT = dithiothreitol; EBV = Epstein-Barr virus; EDTA = ethylenediaminetetraacetic acid; HBc = hepatitis B core; HLA-DRB1 = human leukocyte antigen-DRB1; HV3 = the third allelic hypervariable region; *IC*_50 _= half inhibitory concentration; MHC = major histocompatibility complex; NO = nitric oxide; NOS = nitric oxide synthase; PBS = phosphate-buffered saline; PKA = protein kinase A; RA = rheumatoid arthritis; ROS = reactive oxygen species; SE = shared epitope; TBE = Tris borate-ethylenediaminetetraacetic acid.

## Competing interests

SL and JH are named co-inventors on US patent 7,074,893, owned by the Regents of the University of Michigan. The other authors declare that they have no competing interests.

## Authors' contributions

SL performed signal transduction experiments and analyzed the data. ZL performed some of the experiments involving cAMP and PKA signaling. OB prepared recombinant HBc particles. LX performed PKA activation experiments in EBV-transformed B cells. PP designed HBc particles and participated in data analysis. JH conceived of and designed the study and reviewed the analyzed data. All authors read and approved the final manuscript.

## Supplementary Material

Additional file 1 A file containing Supplemental figure 1. Click here for file

Additional file 2 A file containing Supplemental figure 2. Click here for file

## Figures and Tables

**Table 1:** Peptides used in this study

Designation	Amino acid sequence	Encoded by *HLA-DRB1 *alleles
65–79*0401	KDLLEQKRAAVDTYC	*0401, *0413, *0416, *0421, *1419, *1421
65–79*0402	KDILEDERAAVDTYC	*0402, *0414, *0103, *1102, *1116, *1120, *1121, *1301, *1302, *1304, *1308, *1315, *1315, *1317, *1319, *1322, *1416
65–79*0403	KDLLEQRRAEVDTYC	*0403, *0406, *0407, *0417, *0420
65–79*0404	KDLLEQRRAAVDTYC	*0404, *0405, *0408, *0410, *0419, *0101, *0102, *1402, *1406, *1409, *1413, *1417, *1420
65–79*1001	KDLLERRRAAVDTYC	*1001
Pentapeptide		
1	QKRAA	*0401, *0413, *0416, *0421, *1419, *1421
2	QRRAA	*0404, *0405, *0408, *0410, *0419, *0101, *0102, *1402, *1406, *1409, *1413, *1417, *1420
3	DERAA	*0402, *0414, *0103, *1102, *1116, *1120, *1121, *1301, *1302, *1304, *1308, *1315, *1315, *1317, *1319, *1322, *1416
4	RRRAA	*1001
5	RRRAE	*09, *1401, *1404, *1405, *1407, *1408, *1410, *1411, *1414, *1418
6	DRRAA	*1601, *1602, *1605, *0415, *11011, *11012, *11041, *11042, *1105, *1106, *11081, *11082*1109, *1110, *1112, *1115, *1118, *1119, *1122, *1201, *12021, *12022, *12031, *12032, *1305, *1306, *1307, *1311, *1312, *1314, *1321, *0805
7	QARAA	*15, *1309
8	QKRGR	*03, *0422, *1107
9	QKRLA	Not applicable (modified peptide)
10	QKRAE	Not applicable (modified peptide)
11	QKCLA	Not applicable (modified peptide)
12	QECLA	Not applicable (modified peptide)
13	DKCLA	Not applicable (modified peptide)

A, alanine; C, cysteine; D, aspartic acid; E, glutamic acid; G, glycine; *HLA-DRB1*, human leukocyte antigen-DRB1; I, isoleucine; K, lysine; L, leucine; Q, glutamine; R, arginine; T, threonine; V, valine; Y, tyrosine.
