# Supplementary material for: The rheumatoid arthritis shared epitope increases cellular susceptibility to oxidative stress by antagonizing an adenosine-mediated anti-oxidative pathway
Source: Arthritis Res Ther. 2007 Jan 25;9(1):R5. doi: 10.1186/ar2111 (PMC1865041; doi:10.1186/ar2111)
Supplement: Additional file 1 — A file containing Supplemental figure 1. [file ar2111-S1.pdf]

Supplemental Figure 1

A

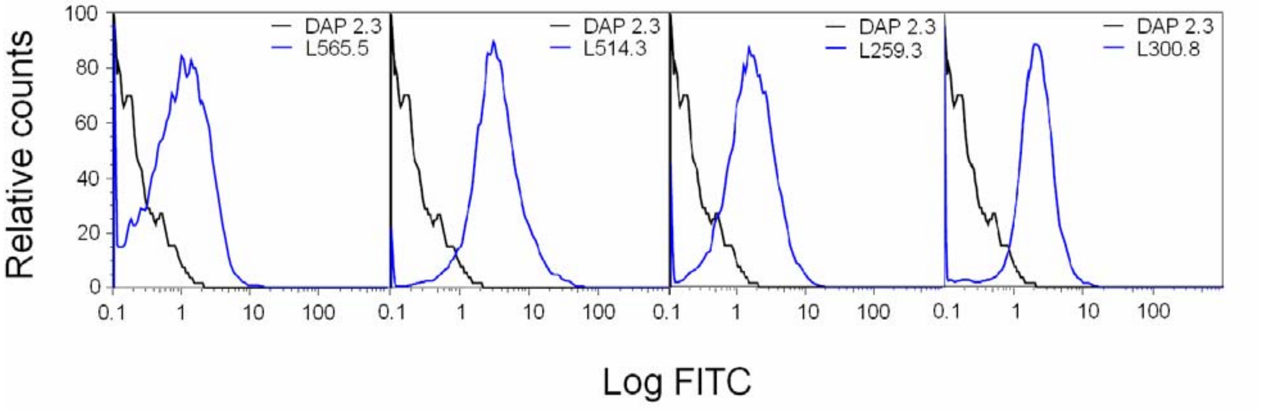

B

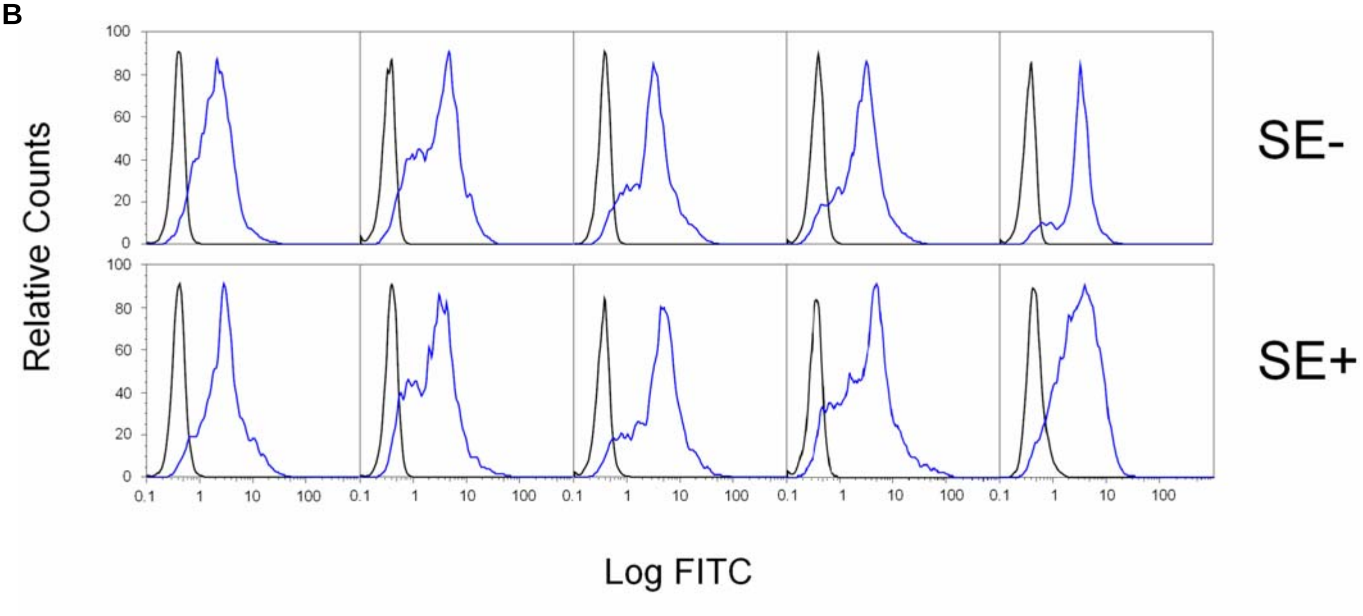

Supplemental Figure 1. **A**, different L cell lines were incubated for 1 hr at 4°C with FITC-conjugated mouse anti-HLA-DR antibody (Biosource) at a 1:100 dilution. Cells were washed twice and fixed with 1% formaldehyde. Fluorescence was measured using a Coulter Epics XL Cytometer. As can be seen, all transfectants (blue line) had equivalent levels of HLA-DR surface expression. Untransfected L cells (black line) showed negative staining. In **B**, Five randomly selected SE-negative EBV transformed B cell lines (upper panel) and 5 randomly selected SE-positive EBV transformed B cell lines (lower panel) were stained with either FITC-conjugated mouse anti- HLA-DR antibody as in **A** (blue line), or with isotype-matched control antibody (black line). As can be seen, SE-positive and SE-negative cells showed equivalent surface expression of HLA-DR molecules.
