# Supplementary material for: The rheumatoid arthritis shared epitope increases cellular susceptibility to oxidative stress by antagonizing an adenosine-mediated anti-oxidative pathway
Source: Arthritis Res Ther. 2007 Jan 25;9(1):R5. doi: 10.1186/ar2111 (PMC1865041; doi:10.1186/ar2111)
Supplement: Additional file 2 — A file containing Supplemental figure 2. [file ar2111-S2.pdf]

Supplemental Figure 2

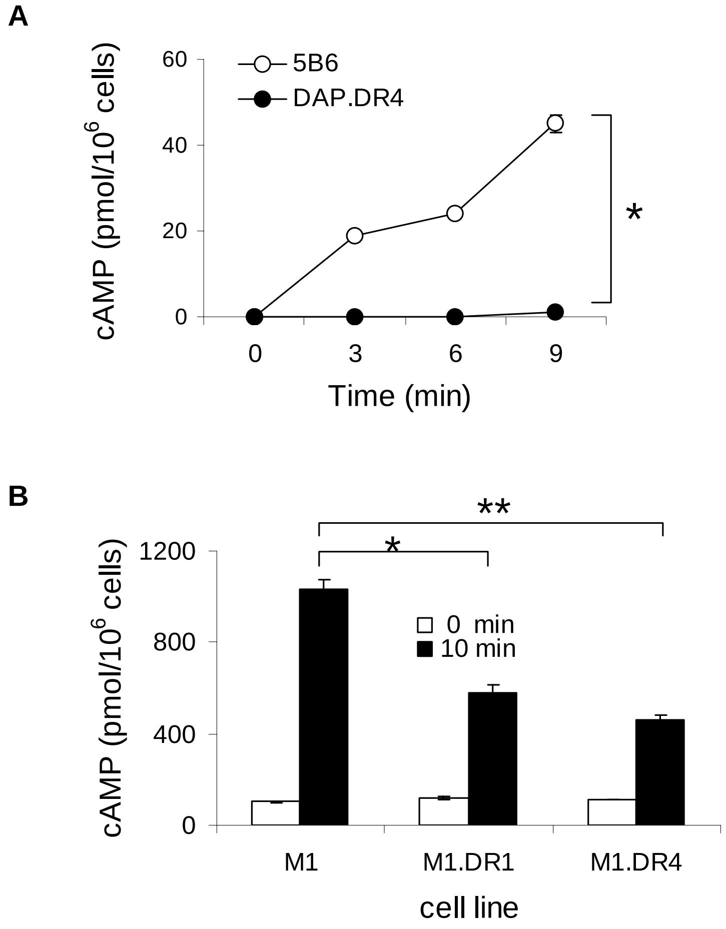

Supplemental Figure 2. A, cAMP levels in L cell transfectants were measured at different times following stimulation with 25 μM forskolin. As can be seen, 5B6 cells (L cells transfected with a vector containing the DRα sequence only, which does not allow surface expression of the DR molecule) had an intact cAMP response, while DAP.DR4 (L cell transfected with a vector encoding both the DRα and DRβ*0401 sequences) showed no response. *****, $p < 0.05$. **B**. Untransfected M1 cells (M1), M1 cell transfectants expressing on their surface the DRβ*0101 (M1.DR1), or the DRβ*0401 (M1.DR4) molecules were stimulated for 10 min with 25 μM forskolin and cAMP levels were measured. As can be seen, the SE-positive M1.DR1 and M1.DR4 lines had a markedly blunted cAMP response. ***,** $p <$ 0.05; **, $p < 0.01$.
